# Correction: Polydiacetylene-based colorimetric and fluorometric sensors for lead ion recognition

**DOI:** 10.1039/d4ra90069c

**Published:** 2024-06-06

**Authors:** Xipeng Chen, Yang Li, Yalin Yang, Yuchuan Dong, Jinwen Guo, Shu-Wei Chen, Jinyi Wang

**Affiliations:** a College of Chemistry & Pharmacy, Northwest A&F University Yangling Shaanxi 712100 P. R. China chenshuwei2011@nwsuaf.edu.cn jywang@nwsuaf.edu.cn

## Abstract

Correction for ‘Polydiacetylene-based colorimetric and fluorometric sensors for lead ion recognition’ by Xipeng Chen *et al.*, *RSC Adv.*, 2022, **12**, 22210–22218, https://doi.org/10.1039/D2RA03435B.

The authors regret that the order of authors in the author list was shown incorrectly in the original article. The corrected author list is as shown above.

The Royal Society of Chemistry apologises for these errors and any consequent inconvenience to authors and readers.

## Supplementary Material

